# Circulating metabolites and the development of type 2 diabetes in Chinese adults

**DOI:** 10.2337/dc21-1415

**Published:** 2022-02-01

**Authors:** Fiona Bragg, Christiana Kartsonaki, Yu Guo, Michael Holmes, Huaidong Du, Canqing Yu, Pei Pei, Ling Yang, Donghui Jin, Yiping Chen, Dan Schmidt, Daniel Avery, Jun Lv, Junshi Chen, Robert Clarke, Michael Hill, Liming Li, Iona Millwood, Zhengming Chen

**Affiliations:** 1 Clinical Trial Service Unit & Epidemiological Studies Unit (CTSU), Nuffield Department of Population Health, University of Oxford, Oxford, UK; 2 Medical Research Council Population Health Research Unit, Nuffield Department of Population Health, University of Oxford, Oxford, UK; 3 Fuwai Hospital Chinese Academy of Medical Sciences, National Center for Cardiovascular Diseases, Beijing, China; 4 Department of Epidemiology & Biostatistics, School of Public Health, Peking University Health Science Center, Beijing, China; 5 Peking University Center for Public Health and Epidemic Preparedness & Response, Beijing, China; 6 Chinese Academy of Medical Sciences, Beijing 102308, China; 7 Hunan Centre for Disease Control and Prevention, Furong Mid Road, Changsha, Hunan, China; 8 China National Center For Food Safety Risk Assessment, Beijing, China

## Abstract

**Objective:**

To assess prospective associations of circulating metabolites with risk of type 2 diabetes (T2D) among Chinese adults.

**Research Design and Methods:**

A case-cohort study within the 8-year prospective China Kadoorie Biobank comprised 882 incident cases of T2D and 789 subcohort participants. NMR metabolomic profiling quantified 225 metabolites in stored baseline plasma samples. Cox regression related individual metabolites with T2D risk, adjusting for potential confounders and fasting time.

**Results:**

After correction for multiple testing, 163 metabolites were significantly associated with risk of T2D (*p*<0.05). There were strong positive associations of VLDL particle size, the ratio of apolipoprotein B/apolipoprotein A1, branched chain amino acids, glucose and triglycerides with T2D, and inverse associations of HDL-cholesterol, HDL particle size, and relative omega-3 and saturated fatty acid concentrations.

**Conclusions:**

In Chinese adults, metabolites across diverse pathways were independently associated with T2D risk, providing valuable aetiological insights and potential to improve T2D risk prediction

## Introduction

Understanding of type 2 diabetes (T2D) aetiological pathways is fundamental to disease prevention and development of new therapies. Comprehensive profiling of circulating metabolites in diverse populations is essential for improved understanding of the molecular basis of T2D. With the largest T2D population globally,^1^ and with marked differences in genetic, environmental and lifestyle factors, when compared with more widely-studied Western populations, large-scale investigation of T2D-associated metabolomic profiles in the Chinese population is needed.

## Research Design and Methods

The China Kadoorie Biobank (CKB) includes 512,715 adults aged 30-79 years, who were recruited from 10 areas of China in 2004-2008.^2^ The baseline survey collected detailed information on medical history, sociodemographic and lifestyle factors, and physical measurements. Venous blood samples were collected (with time since last food recorded) for immediate on-site measurement of random plasma glucose (RPG) concentrations (SureStep Plus system, LifeScan, CA, USA) and those with a RPG ≥140 mg/dL and <200 mg/dL were invited for fasting glucose testing the following day. Remaining blood samples were retained for long-term storage. Participants were followed up through on-going linkage to death and disease (including diabetes) registries and to the national health insurance system, providing data on ICD-10 coded morbidity and mortality. Local, national and international ethics approval was obtained. All participants provided written informed consent.

We selected, using simple random sampling, 900 from 7721 incident T2D cases (ICD-10 E11) recorded by 1 January 2017 (median [IQR] 8.6 [5.2] years follow-up) and without self-reported or newly-diagnosed diabetes^3^ at baseline, and a subcohort of 905 participants from 31,443 randomly selected genotyped participants. The main analyses included 882 T2D cases and a subcohort of 789 (including 26 with T2D) after excluding participants with inadequate plasma samples or with case status mismatch or diabetes at baseline.

NMR-based profiling quantified 225 metabolites (directly-measured or ratios of these) in baseline plasma samples.^4^ Cox proportional hazards models, using the Prentice pseudo-partial likelihood^5^ and correcting for multiple testing,^6^ were fitted to estimate T2D hazard ratios (HRs) for metabolites, adjusting for sociodemographic and lifestyle factors, fasting time, adiposity and family history of diabetes. Metabolites were examined as categorical (split at quartiles) and continuous (per 1-SD increment) variables. Additional analyses further adjusted for RPG.

## Results

At recruitment, T2D cases were, on average, older than subcohort participants (55.1 [SD 9.6] vs. 51.9 [10.6] years), had higher adiposity levels (BMI: 25.7 [3.6] vs. 23.9 [3.6] kg/m^2^; WC: 85 [10] vs. 80 [10] cm), and were more likely to have a family history of T2D (10% vs. 8%). Use of lipid-lowering medication was rare (<1%). Overall, 163 of the 225 quantified metabolite measures were independently associated with T2D (*p*<0.05), with continuous, largely linear, relationships. Following additional adjustment for RPG, 147 significant associations remained.

**Figure 1:**
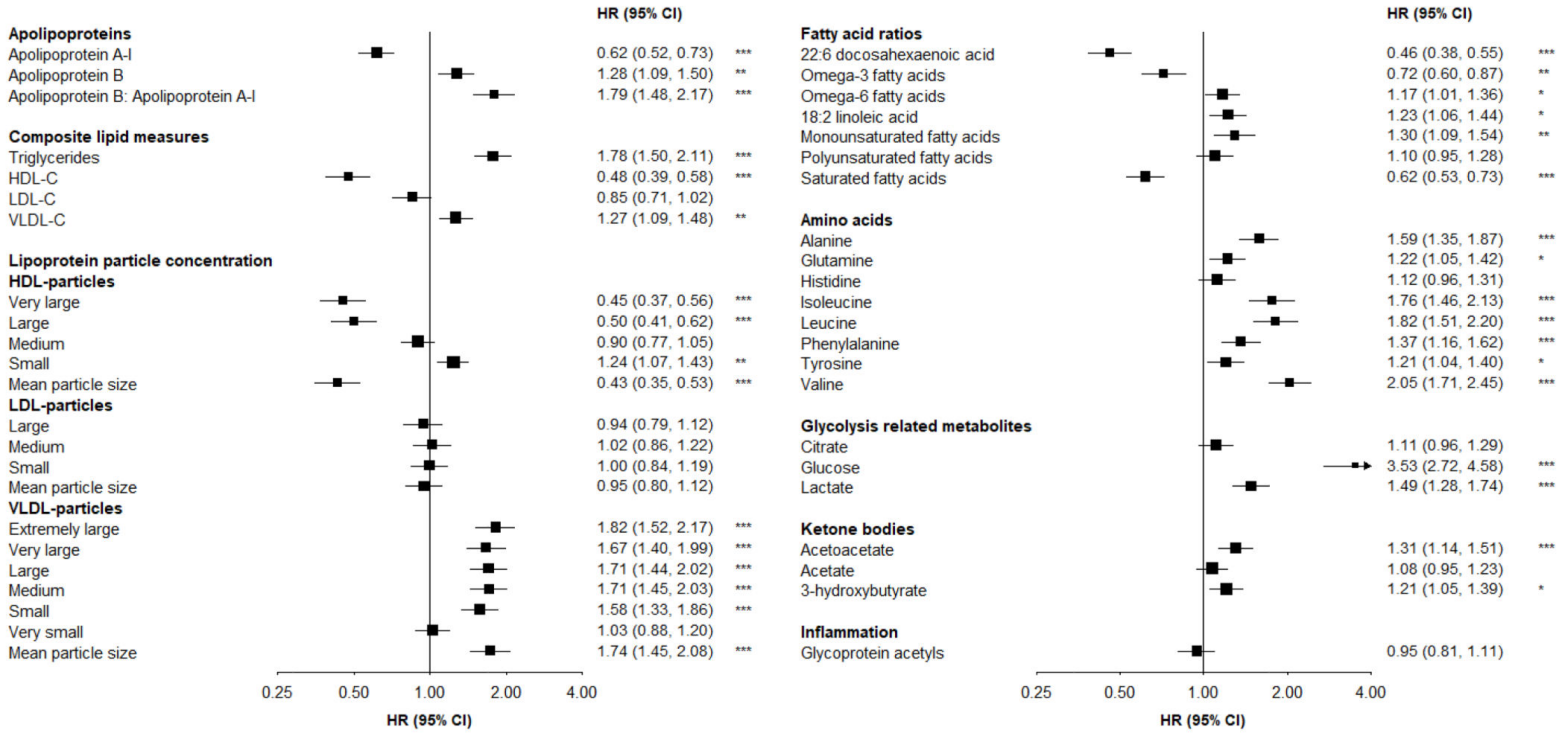
Associations of circulating metabolites with risk of incident type 2 diabetes (n=882), adjusted for fasting time Adjusted for age (numeric), sex, study area (10 areas), education (6 categories), fasting time (numeric), smoking (ever regular vs. other), alcohol drinking (ever regular vs. other), physical activity (metabolic equivalent of task hours per day, numeric), dietary factors (frequency of consumption of meat, fish, fresh fruit, dairy products; 4 times/week or more vs. other), family history of diabetes (any first degree relative vs. none), body mass index (numeric) and waist circumference (numeric). Squares represent the HR per 1-SD higher metabolite. Horizontal lines represent the corresponding 95% CI. Fatty acid ratios represent ratios of individual to total fatty acids. *p≤0.05, **p≤0.01, ***p≤0.001 after adjustment for multiple testing using Benjamini-Hochberg correction.

There were strong positive associations of apolipoprotein B:apolipoprotein A-1 (HR=1.79 per 1-SD higher) and triglyceride concentrations (1.78) with T2D, and a weaker positive association of VLDL-cholesterol concentration (1.27) (Figure 1). Higher HDL-cholesterol concentrations were associated with lower T2D risk (0.48). Each 1-SD increment in mean VLDL and HDL particle sizes were associated with 74% higher and 57% lower risks of T2D, respectively.

Leucine and isoleucine were both strongly positively associated with T2D (~80% higher risk per 1-SD), as was valine (HR=2.05). There were weaker positive associations of aromatic amino acids (phenylalanine:1.37; tyrosine: 1.21), alanine (1.59) and glutamine (1.22). Higher relative concentrations of total omega-3 fatty acids were associated with lower T2D risk (0.72), with a particularly strong inverse association (0.46) of docosahexaenoic acid. In contrast, each 1-SD increment in total omega-6 fatty acids was associated with 17% higher T2D risk. There were positive and inverse associations, respectively, of monounsaturated (1.30) and saturated (0.62) fatty acids. Lactate, acetoacetate and 3-hydroxybutyrate were modestly positively associated with T2D (1.49, 1.31 and 1.21, respectively), but there was no association of glycoprotein acetyls.

With the exception of moderately stronger associations for lipoprotein measures among younger participants, no marked differences in the associations of metabolites were observed by age, sex or urban/rural residence, and associations remained largely unchanged following exclusion of the first 2 years’ follow-up.

## Conclusions

This is the largest prospective investigation in China of the associations of diverse circulating metabolites with T2D risk. Among relatively lean Chinese adults, large numbers of metabolites, across varied pathways, were found to be independently associated with T2D. Furthermore, the majority of these associations persisted after adjustment for glycaemia.

The lipid and lipoprotein profile associated with higher T2D risk in the present study, including higher concentrations of VLDL-cholesterol and triglycerides, lower concentrations of HDL-cholesterol, and smaller mean HDL particle and larger mean VLDL particle sizes, broadly replicates previous findings.^7^ However, the current associations are moderately more extreme than those previously observed, for example, in a cohort of ~12,000 young adults in Finland using the same NMR-metabolomics platform.^7^ The focus of the metabolomics platform used on lipid and lipoprotein measures, and correlation between these, partly explains the large number of significant associations observed. However, metabolites associated with T2D were diverse, and included branched chain amino acids (BCAA)—leucine, isoleucine and valine—the associations of which were among the strongest observed. This is consistent with previous investigations,^8,9^ and with genetic association studies suggesting higher BCAA concentrations result from insulin resistance and, in turn, cause T2D.^10,11^ The observed lipid and lipoprotein profile is also consistent with the importance of insulin resistance as a precursor of T2D,^12^ even in comparatively lean populations such as in CKB.

The higher risks of T2D with higher concentrations of alanine, phenylalanine and tyrosine reflect well-established associations.^7–9^ We also observed higher T2D risk among participants with higher glutamine concentrations, in contrast to other study findings showing null^9^ or even inverse^7^ associations. Further larger studies are needed to clarify this relationship. Although consistent with previous findings in predominantly European population studies,^13^ the notably lower risks of T2D among individuals with lower relative total omega-3 fatty acid and docosahexaenoic acid concentrations represent the first large-scale evidence of such associations in a Chinese population.

Our study has several strengths, including large sample size, prospective design, investigation of metabolite-T2D associations in a comparatively understudied population, and use of a validated NMR platform.^14^ Moreover, the infrequent use of lipid-lowering medications in CKB reduces treatment-associated biases. The study also has limitations. Incident T2D comprised diagnosed cases only, and resulting misclassification would likely underestimate metabolite-associated risks. A similar effect may have resulted from reliance on single metabolite measures, precluding adjustment for intra-individual variation. Furthermore, the targeted NMR platform used is not comprehensive. However, the strong focus on lipids and lipoproteins is clearly relevant to cardiometabolic disorders such as T2D. Finally, use of non-fasting blood samples would be expected to increase inter-individual variation in metabolite concentrations. However, all analyses adjusted for fasting time, and additional adjustment for dietary variables did not appreciably alter risk estimates.

In summary, our study shows that, among relatively lean Chinese adults, a diverse range of circulating metabolites are independently associated with T2D risk. These findings highlight the value of NMR metabolomic profiling for advancing understanding of molecular derangements associated with T2D, with potential translational relevance for enhanced T2D prediction and, ultimately, prevention. Moreover, they may inform selection of metabolites for investigation in future studies to establish causality (e.g., Mendelian randomisation studies), facilitating discovery of novel therapeutic targets.
